# Direct medical costs and length of stay associated with postoperative systemic infectious complications after gynecologic surgery: a propensity score–matched case–control study

**DOI:** 10.3389/fpubh.2026.1796203

**Published:** 2026-03-20

**Authors:** Yan Luo, Wei Li, Lan Chen, Zheng Xiang, Qun Sun, Yujun He, Na Xiao, Qian Xiang

**Affiliations:** 1 Sichuan Academy of Medical Sciences & Sichuan Provincial People’s Hospital, School of Medicine, University of Electronic Science and Technology of China, Chengdu, Sichuan, China; 2 Chengdu Institute of Food Inspection, Irradiation Preservation Key Laboratory of Sichuan Province, Chengdu, Sichuan, China; 3 903 Hospital, Mianyang, Sichuan, China

**Keywords:** case–control study, direct medical costs, doubly robust estimation, economic burden, gynecologic surgery, length of stay, postoperative systemic infectious complications, propensity score matching

## Abstract

**Background and aim:**

To quantify the independent impact of postoperative systemic infectious complications (PSIC) on direct medical costs and length of stay (LOS) for patients undergoing gynecologic surgery.

**Methods:**

This was a retrospective case–control study conducted at a large tertiary healthcare facility in southwestern China. The participants included patients who underwent gynecologic surgery between January 2022 and December 2024. The study included 202 patients with PSIC (case group) and 600 matched patients without PSIC (control group) following 1:3 propensity score matching (PSM). The primary outcomes measured were direct medical costs and LOS, analyzed via a doubly robust estimation framework using Generalized Linear Models (GLMs) to calculate the Average Marginal Effect (AME).

**Results:**

After matching, baseline covariates were largely balanced, with any residual confounding addressed in downstream models. Total unadjusted direct medical costs were significantly higher in the PSIC group compared with the control group (median ¥31,124.06 vs. ¥20,726.43, *p* < 0.001), with a median difference of ¥8,127.02. Patients with PSIC had a significantly longer median overall LOS (12 vs. 8 days, *p* < 0.001) and postoperative LOS (9 vs. 5 days, p < 0.001). Crucially, after doubly robust covariate adjustment, PSIC was found to independently drive an absolute incremental burden of ¥8,960.74 in total medical costs and 4.20 days in prolonged hospitalization (both *p* < 0.001).

**Conclusion:**

PSIC following gynecologic surgery independently and substantially increases direct medical costs and prolongs hospitalization. These findings highlight the significant economic burden of these complications and underscore the value of investing in effective infection prevention strategies to optimize resource utilization.

## 1 Introduction

Gynecologic surgery is a core component of women’s healthcare (1). Globally, the volume of gynecologic surgery is substantial, with an estimated 5 million hysterectomies completed in 2021, including 2.82 million in China (2, 3). Concurrently, the proportion of minimally invasive gynecologic surgery continues to grow (4).

Postoperative infection is a deleterious complication, endangering patients and imposing major economic burdens (5). US data show that surgical site infection (SSI) after hysterectomy can nearly triple hospital costs and markedly increase readmissions (6). Similarly, a UK study demonstrated that SSI significantly extends hospitalization and increases costs, with deep infections further elevating mortality risks (7).

Most research has focused on SSI, whereas postoperative systemic infectious complications (PSIC) remain understudied, despite their likely higher mortality and intensive care demand. Quantitative estimates of the economic burden of PSIC in gynecologic surgery are lacking.

To strictly control for baseline confounding and isolate the absolute incremental impact of PSIC on direct medical costs and length of stay (LOS), we employed a double-robust estimation framework combining propensity score matching (PSM) with generalized linear models (GLMs). These quantitative findings may inform targeted infection prevention strategies and value-based resource allocation, particularly amid China’s shift to Diagnosis Related Groups/Diagnosis Intervention Packet (DRG/DIP)–based payment models.

## 2 Materials and methods

### 2.1 Study design and participants

This retrospective case–control study was conducted at a large tertiary healthcare facility in southwestern China (>6,000 gynecologic surgeries/year). We analyzed data from the Department of Gynecology surgical database (January 2022–December 2024). Potential confounding was addressed by 1:3 nearest neighbor PSM with a caliper width of 0.02. Inclusion criteria were age ≥18 years and complete data records; procedures outside the operating room were excluded. The study adhered to institutional and national ethical standards.

### 2.2 Variable definitions

#### 2.2.1 Postoperative systemic infectious complications (PSIC)

We defined PSIC as a syndromic outcome based on the Chinese National Diagnostic Criteria for Nosocomial Infections. Patients were cases if they met the following within 30 days post-surgery: (1) Abnormal temperature (>38.0 °C or <36.0 °C) for ≥2 days; (2) Systemic manifestations of septicemia (e.g., toxic signs, hypotension, unexplained leukocytosis); (3) Initiation or escalation of systemic therapeutic antibiotics; and (4) At least one blood culture drawn (8, 9). This definition captures both culture-positive and culture-negative significant infections. While this syndromic category may inadvertently capture some non-infectious postoperative systemic inflammatory response syndromes (SIRS), from a health economics perspective, any clinical deterioration severe enough to trigger blood culture orders and antibiotic escalation imposes a tangible, identical resource burden. Thus, it aligns directly with our objective of quantifying real-world medical costs.

#### 2.2.2 Outcome variables

Direct medical costs included all electronic medical record (EMR) documented expenses: service, diagnostic, therapeutic, material, pharmaceutical, and other costs. Length of stay (LOS) was subdivided into length of preoperative preparation stay (LPPS) and length of postoperative stay (LPOS).

#### 2.2.3 Additional clinical definitions

Incision types were classified according to standard surgical wound criteria (Type I: clean, Type II: clean-contaminated, Type III: contaminated). Prophylactic antimicrobial use was defined as adherence to guidelines, specifically the administration of antibiotics within 30–60 min prior to surgical incision. Vaginal preparation involved standardized preoperative douching or scrubbing with antiseptic solutions (e.g., povidone-iodine) performed by nursing staff. An intraoperative blood loss recorded as 0 mL denotes minimal or negligible bleeding rather than an absolute absence of blood. Consequently, for statistical analysis, intraoperative blood loss was stratified into three categories: low (≤50 mL), moderate (50–200 mL), and high (>200 mL).

### 2.3 Statistical analyses

Analyses were performed using R version 4.4.1. We balanced covariates using a multivariable logistic regression PSM model (standardized mean difference (SMD) < 0.1 indicating optimal balance) that incorporated all baseline variables (including demographics, comorbidities, labs, surgical factors). Continuous variables in the matched cohort were compared using Mann–Whitney U tests due to their non-normal distributions. To robustly quantify the absolute incremental medical costs and length of stay purely attributable to PSIC, we employed a double-robust estimation framework on the entire matched cohort. Specifically, Generalized Linear Models (GLMs) were constructed. A GLM with a Gamma distribution and log-link function was utilized for total medical costs to account for right-skewed data, while a Quasi-Poisson GLM with a log-link function was applied for length of stay to handle overdispersion. Both models were further adjusted for specific covariates (including age, BMI, tumor nature, surgical complexity, and operation duration) to eliminate any residual confounding post-PSM. The absolute incremental burden was calculated as the Average Marginal Effect (AME). Significance was set at *p* < 0.05.

## 3 Results

### 3.1 Participant selection and matching

**Figure 1:**
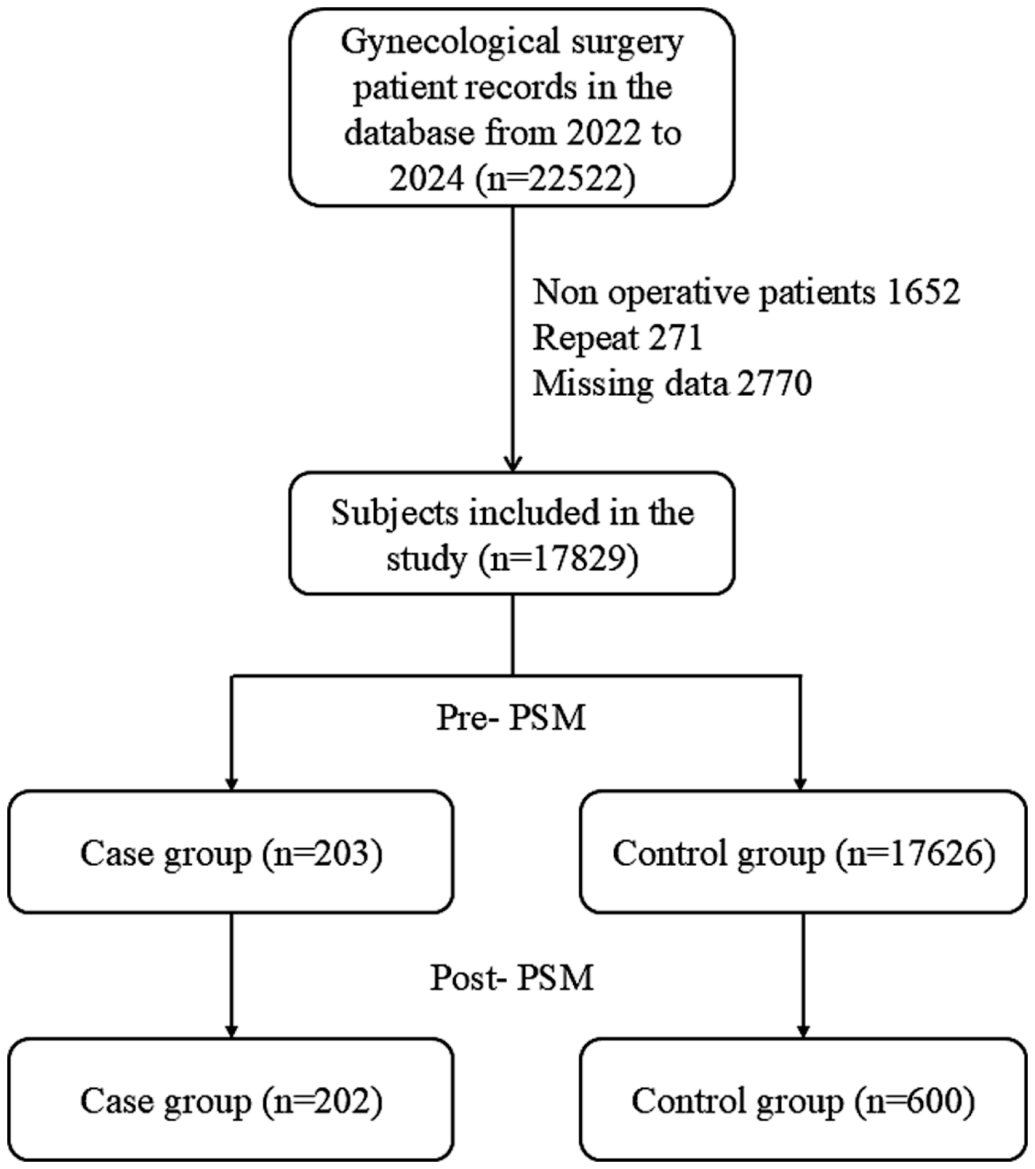
Flow chart of participant selection.

**Figure 2:**
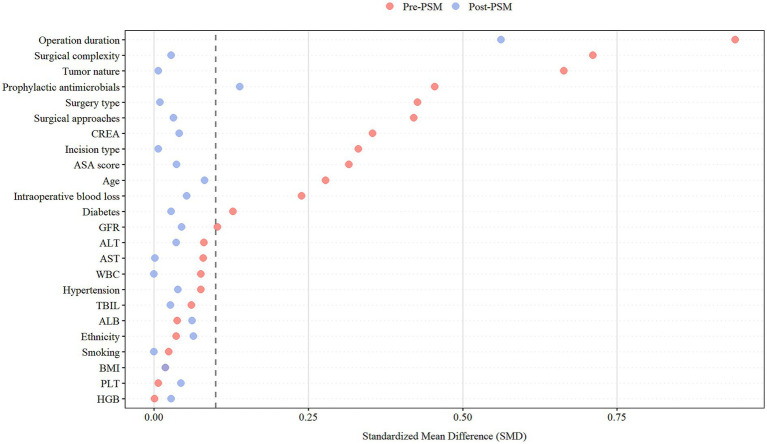
Standardized mean differences between the case and control groups before and after propensity score matching.

**Figure 3:**
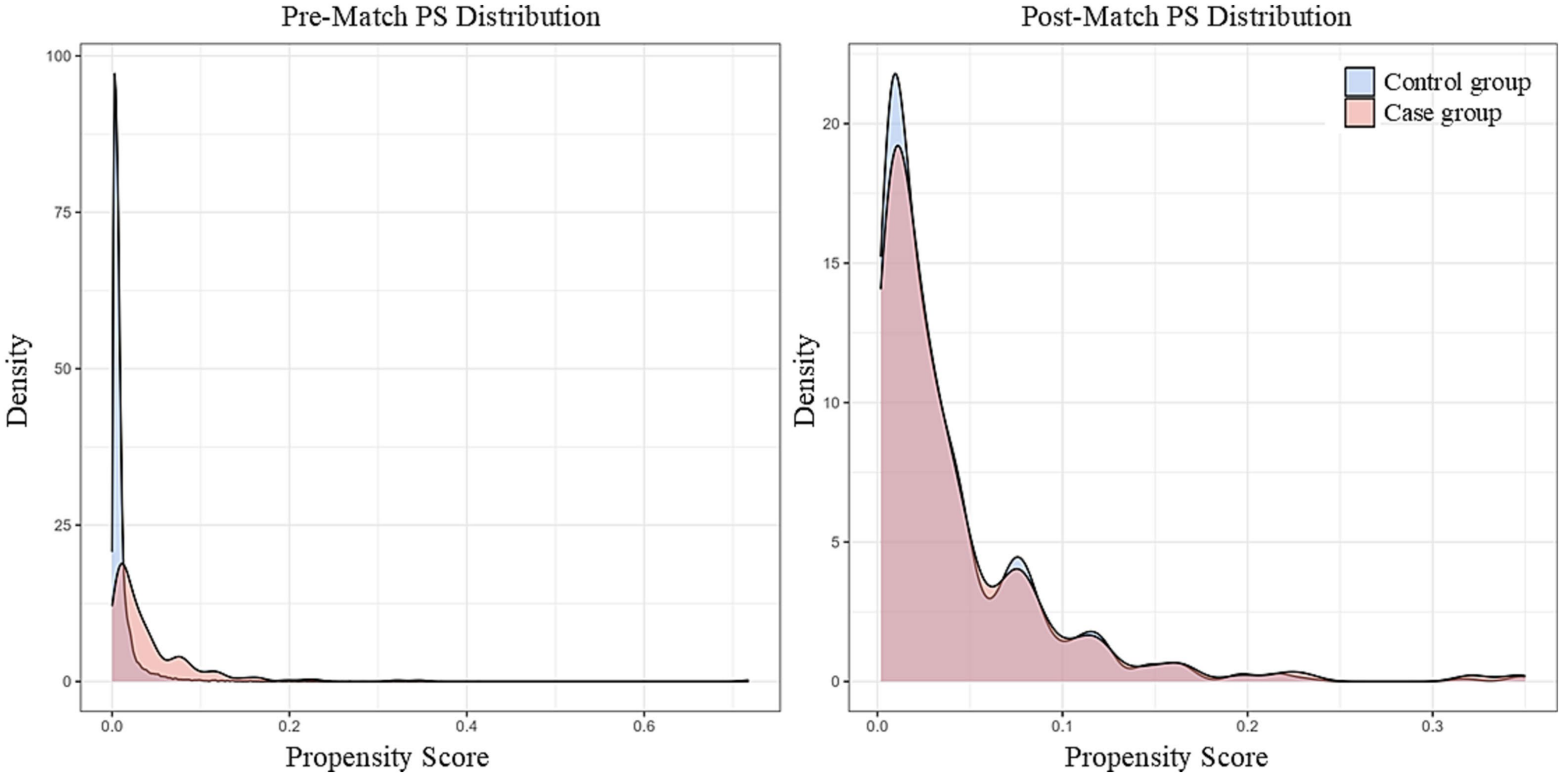
Distribution of propensity scores in the case and control groups before and after matching.

After screening and PSM, 202 PSIC cases and 600 controls were included (Figure 1). Among the initial cases, 25 (12.3%) had positive blood cultures, while 178 (87.7%) met syndromic criteria. Before matching, groups differed significantly in age, diabetes, liver/renal function, and surgical factors (Table 1). After PSM, the vast majority of baseline characteristics achieved optimal balance (SMD < 0.1, Figure 2), with overlapping propensity score distributions (Figure 3). However, a few specific variables, notably operation duration (SMD = 0.562) and the use of prophylactic antimicrobials (SMD = 0.139), retained some degree of imbalance. These residual confounders were fully accounted for in the downstream doubly robust GLMs.

**Table 1:** Baseline characteristics of the case and control groups before and after propensity score matching.

Variables	Pre-PSM			Post-PSM		
	Case group	Control group	SMD	Case group	Control group	SMD
*n*	203	17,626		202	600	
Demographic characteristics						
Ethnicity			0.036			0.064
Han	185 (91.1%)	15,884 (90.1%)		185 (91.6%)	560 (93.3%)	
Zang	12 (5.9%)	1,261 (7.2%)		11 (5.4%)	28 (4.7%)	
Others	6 (3.0%)	481 (2.7%)		6 (3.0%)	12 (2.0%)	
Age (years)	49.00 (40.0–56.0)	45.00 (35.0–52.0)	0.278	49.00 (40.00–55.75)	49.00 (40.00–58.25)	0.082
BMI (kg/m^2^)	22.83 (20.76–25.10)	22.66 (20.70–24.97)	0.019	22.83 (20.73–24.98)	22.89 (20.95–25.21)	0.019
Smoking	0 (0.0%)	10 (0.1%)	0.024	0 (0.0%)	0 (0.0%)	0.000
Preexisting comorbidities						
Diabetes	15 (7.4%)	712 (4.0%)	0.128	15 (7.4%)	49 (8.2%)	0.028
Hypertension	27 (13.3%)	1891 (10.7%)	0.076	27 (13.4%)	89 (14.8%)	0.039
Preoperative laboratory tests						
WBC (10^9^/L)	9.69 (7.08–12.10)	9.37 (7.55–11.17)	0.076	9.69 (7.11–12.16)	9.49 (7.39–11.62)	0.000
HGB (g/L)	106.0 (85.5–125.0)	117.0 (106.0–127.0)	0.001	106.0 (85.25–125.0)	115.0 (98.0–126.0)	0.028
PLT (10^9^/L)	201.0 (159.0–258.0)	209.0 (177.0–244.0)	0.007	200.5 (159.0–256.7)	205.0 (165.0–245.0)	0.044
CREA (μmol/L)	72.16 (61.0–92.0)	81.05 (71.7–93.5)	0.354	72.25 (61.0–92.5)	77.45 (61.7–92.8)	0.041
GFR (ml/min/1.73m^2^)	106.6 (97.3–115.7)	108.4 (103.0–114.1)	0.103	106.8 (97.3–115.7)	106.5 (97.1–113.2)	0.045
ALT (U/L)	14.0 (10.0–21.0)	15.0 (12.0–20.0)	0.081	14.0 (10.0–20.75)	15.0 (11.0–20.25)	0.036
AST (U/L)	22.0 (17.0–27.0)	22.0 (19.0–25.0)	0.080	22.0 (17.0–27.0)	22.0 (18.0–28.0)	0.002
TBIL (μmol/L)	11.40 (8.05–16.50)	13.20 (10.90–15.80)	0.061	11.40 (8.03–16.45)	13.20 (9.47–16.60)	0.027
ALB (g/L)	41.20 (34.9–45.9)	43.90 (42.1–45.6)	0.038	41.20 (35.0–45.9)	43.55 (36.6–46.3)	0.062
Surgery related information						
Tumor nature			0.664			0.007
Benign	122 (60.1%)	16,322 (92.6%)		122 (60.4%)	364 (60.7%)	
Malignant	81 (39.9%)	1,304 (7.4%)		80 (39.6%)	236 (39.3%)	
Surgical complexity			0.711			0.028
Routine	67 (33.0%)	11,707 (66.4%)		67 (33.2%)	193 (32.2%)	
Complex/Radical	136 (67.0%)	5,919 (33.6%)		135 (66.8%)	407 (67.8%)	
Surgical approaches			0.421			0.032
Open	62 (30.5%)	1964 (11.1%)		61 (30.2%)	186 (31.0%)	
Laparoscope	141 (69.5%)	15,662 (88.9%)		141 (69.8%)	414 (69.0%)	
Operation duration (min)	150.0 (100–250)	65.0 (25–105)	0.941	150.0 (100–247.5)	100.0 (50–170)	0.562
Intraoperative blood loss			0.239			0.053
Minimal	178 (87.7%)	16,837 (95.5%)		177 (87.6%)	537 (89.5%)	
Moderate	9 (4.4%)	530 (3.0%)		9 (4.5%)	28 (4.7%)	
High	16 (7.9%)	259 (1.5%)		16 (7.9%)	35 (5.8%)	
ASA score			0.316			0.037
I, II	173 (85.2%)	16,996 (96.4%)		173 (85.6%)	527 (87.8%)	
III, IV, V	30 (14.8%)	630 (3.6%)		29 (14.4%)	73 (12.2%)	
Incision type			0.331			0.007
I, II	11 (5.4%)	2,276 (12.9%)		11 (5.4%)	34 (5.7%)	
III	192 (94.6%)	15,350 (87.1%)		191 (94.6%)	566 (94.3%)	
Surgery type			0.427			0.010
Uterine	79 (38.9%)	10,528 (59.7%)		79 (39.1%)	238 (39.7%)	
Ovarian & adnexal	74 (36.5%)	4,482 (25.4%)		73 (36.1%)	174 (29.0%)	
Vulvar & cervical	49 (24.1%)	1932 (11.0%)		49 (24.3%)	187 (31.2%)	
Other	1 (0.5%)	684 (3.9%)		1 (0.5%)	1 (0.2%)	
Prophylactic antimicrobials	160 (78.8%)	10,614 (60.2%)	0.455	160 (79.2%)	509 (84.8%)	0.139

Values are presented as *n* (%) or median (IQR). SMD, standardized mean difference. A post-PSM SMD < 0.1 indicates optimal balance between groups. Surgeon ID was also included in the propensity score matching algorithm to control for provider-level variations, but is omitted from this table for brevity. Post-PSM covariates that remained slightly imbalanced (e.g., prophylactic antimicrobials) or specific intraoperative variables (e.g., operation duration) were further comprehensively adjusted in the downstream GLM via doubly robust estimation.

### 3.2 Direct medical costs

Total direct medical costs were significantly higher (*p* < 0.001) in the case group (median ¥31,124.06, IQR 20,572.70–44,113.33) compared with controls (median ¥20,726.43, IQR 14,325.91–33,872.40), representing an unadjusted median difference of ¥8,127.02. Itemized costs—including services, diagnostics, therapeutics, pharmaceuticals, materials—were all significantly higher in the case group (all *p* < 0.001; Table 2).

**Table 2:** Comparison of itemized direct medical costs between the case and control groups (CNY).

Medical cost items	Case group (*n* = 202) median (IQR)	Control group (*n* = 600) median (IQR)	Median difference (95% CI)	*p*-value
Service costs (total)	1,060.05 (703.45–1,774.90)	568.50 (364.60–1,022.85)	435.40 (351.40–523.20)	<0.001
Nursing service fees	349.00 (235.25–566.50)	204.50 (137.00–351.25)	133.00 (106.00–163.00)	<0.001
Medical service fees	669.50 (440.00–1,119.70)	359.00 (219.15–660.55)	295.20 (239.20–353.40)	<0.001
Diagnostic costs (total)	5,888.00 (3,752.25–9,538.75)	3,067.50 (2,284.50–5,963.50)	2,254.00 (1,761.00–2,762.00)	<0.001
Pathological diagnostic fees	1,061.00 (481.75–2,533.50)	671.50 (219.00–1,574.00)	330.00 (200.00–492.00)	<0.001
Clinical diagnostic fees	113.00 (82.00–390.50)	97.00 (43.00–210.25)	36.00 (15.00–48.00)	<0.001
Laboratory diagnostic fees	3,063.50 (2,341.00–4,571.00)	1,741.50 (1,419.25–2,195.25)	1,369.00 (1,180.00–1,582.00)	<0.001
Imaging diagnostic fees	1,002.50 (405.00–2,488.75)	496.00 (290.00–1,627.50)	240.00 (144.00–385.00)	<0.001
Therapeutic costs (total)	9,407.70 (7,101.86–13,709.59)	7,179.81 (4,889.67–11,553.51)	2,370.71 (1,641.34–3,106.02)	<0.001
Non-surgical clinical physical therapy fees	403.50 (218.00–941.50)	243.00 (112.00–548.00)	131.00 (80.00–188.00)	<0.001
Rehabilitation fees	0.00 (0.00–114.00)	0.00 (0.00–114.00)	0.00 (0.00–0.00)	0.705
Surgical therapeutic fees	7,821.82 (5,836.44–10,971.97)	6,446.45 (4,300.72–9,475.49)	1,515.80 (960.00–2,083.39)	<0.001
General therapeutic procedure fees	835.50 (531.25–1,522.62)	403.25 (266.88–746.38)	381.50 (313.50–455.00)	<0.001
Pharmaceutical costs (total)	3,175.91 (2,190.93–6,999.59)	1,702.54 (1,146.35–2,812.84)	1,443.55 (1,176.06–1,741.84)	<0.001
Material costs (total)	7,752.24 (5,181.01–12,636.25)	6,963.49 (3,512.07–12,023.45)	1,191.98 (316.44–2,207.60)	0.005
Disposable materials for examinations	25.73 (19.88–97.98)	19.22 (14.87–84.75)	6.78 (4.50–9.50)	<0.001
Disposable materials for surgeries	6,201.56 (3,835.98–10,864.35)	5,667.50 (2,329.23–10,392.71)	901.79 (192.50–1,765.50)	0.010
Disposable materials for treatments	1,279.66 (974.27–1,839.17)	925.82 (560.41–1,434.88)	404.68 (299.87–505.73)	<0.001
Other costs	80.00 (40.00–80.00)	40.00 (40.00–80.00)	0.00 (0.00–0.00)	0.014
Total costs	31,124.06 (20,572.70–44,113.33)	20,726.43 (14,325.91–33,872.40)	8,127.02 (5,832.59–10,592.08)	<0.001

### 3.3 Length of stay

**Figure 4:**
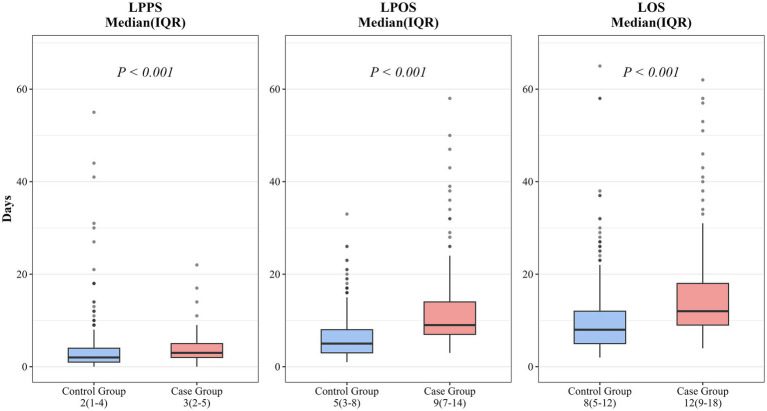
Comparison of the length of preoperative stay (LPPS), length of postoperative stay (LPOS), and total length of stay (LOS) between the case and control groups.

Cases had significantly longer LPOS (9 vs. 5 days, *p* < 0.001) and total LOS (12 vs. 8 days, *p* < 0.001) compared with controls. The length of preoperative stay (LPPS) was also slightly but significantly longer in the case group (3 vs. 2 days, *p* < 0.001), reflecting a potentially more complex preoperative clinical status among patients who eventually developed PSIC (Figure 4).

### 3.4 Adjusted incremental burden attributable to PSIC

To isolate the independent impact of PSIC from any residual confounding factors (such as surgical complexity and operation duration), double-robust GLM estimations were performed (Table 3). After rigorous covariate adjustment, PSIC was independently associated with a 1.34-fold increase in total medical costs (Adjusted Cost Ratio 1.34, 95% CI 1.24–1.46) and a 1.48-fold increase in total LOS (Adjusted Incidence Rate Ratio 1.48, 95% CI 1.37–1.59). By calculating the AME, the absolute incremental burden purely attributable to PSIC was precisely quantified as ¥8,960.74 (95% CI 6,428.93–11,492.55) in excess medical costs and 4.20 days (95% CI 3.36–5.04) in prolonged hospital stay (both *p* < 0.001).

**Table 3:** Double-robust estimation of the incremental medical costs and length of stay attributable to PSIC.

Outcomes	Models	Relative effect (95% CI)^a^	Incremental burden (95% CI)^b^	*p*-value
Total medical costs (CNY)	Unadjusted model^c^	1.50 (1.42–1.58)	8,127.02 (5,832.59–10,592.08)	<0.001
	Adjusted model^d^	1.34 (1.24–1.46)	8,960.74 (6,428.93–11,492.55)	<0.001
Length of stay (Days)	Unadjusted model^c^	1.51 (1.43–1.59)	4.00 (3.00–4.00)^e^	<0.001
	Adjusted model^f^	1.48 (1.37–1.59)	4.20 (3.36–5.04)	<0.001

CI, confidence interval; CNY, Chinese Yuan; GLM, generalized linear model; PSIC, postoperative systemic infectious complications.

a Relative effect represents the Cost Ratio for medical costs and the Incidence Rate Ratio for length of stay, derived from the exponentiated coefficients of the respective models.

b Incremental burden represents the absolute increase in costs (CNY) or length of stay (days) purely attributable to PSIC. For the adjusted models, this was calculated as the AME.

c The unadjusted models represent the crude differences within the propensity score-matched cohort (Case: *n* = 202; Control: *n* = 600) without further covariate adjustment.

d The adjusted model for medical costs was a GLM with a Gamma distribution and log-link function, further adjusting for age, BMI, tumor nature, surgical complexity, and operation duration (Double-robust approach).

e The unadjusted incremental burden for length of stay represents the median difference (Hodges-Lehmann estimator) calculated via the Wilcoxon rank-sum test.

f The adjusted model for length of stay was a GLM with a Quasi-Poisson distribution and log-link function, adjusting for the same covariates as the cost model.

## 4 Discussion

This propensity score–matched case–control study shows that PSIC in gynecologic surgery incurs a significant economic and resource burden. Patients with PSIC incurred substantially higher unadjusted direct medical costs (¥31,124.06 vs. ¥20,726.43, median difference ¥8,127.02, *p* < 0.001). Furthermore, PSIC was univariately associated with a median increase of 4 days in LPOS (9 vs. 5 days) and 4 days in overall LOS (12 vs. 8 days, both p < 0.001). Crucially, after rigorous double-robust covariate adjustment, we determined that PSIC independently drove an absolute incremental burden of ¥8,960.74 and prolonged hospitalization by 4.20 days. To our knowledge, this is the first study to employ a doubly robust framework to quantify the pure burden of PSIC after gynecologic surgery, filling a knowledge gap regarding postoperative infectious complications in this field.

The comprehensive cost increase observed across all expenditure categories reflects the multifaceted resource demands of managing PSIC, including treatment, consumables, diagnostics, medical services, pharmaceuticals, and nursing care. The treatment of PSIC, particularly when bloodstream infection is present, typically necessitates extended courses of antimicrobials, sometimes including costly reserve antibiotics for resistant organisms (10). Diagnostic expenses rise due to repeated blood cultures, additional laboratory tests to monitor organ function and inflammatory markers, and potential imaging examinations to identify metastatic foci of infection (11). The need for more intensive monitoring and management of potential sepsis sequelae further drives up medical service and nursing costs (12). Consumable expenditures increase with extended intravenous access needs and additional wound care supplies (10).

The increase in LOS was predominantly postoperative (9 vs. 5 days, *p* < 0.001). Interestingly, LPPS was also slightly longer in the case group (3 vs. 2 days, *p* < 0.001), reflecting a potentially more complex baseline clinical status among patients prone to infection. However, our double-robust GLM confirmed that despite this baseline complexity, the occurrence of PSIC independently accounted for an additional 4.20 days of hospitalization. Longer LOS incurs substantial direct costs and also has a range of sequelae that are not always captured, such as increased risk of hospital acquired complications (such as pressure injuries and venous thromboembolism), longer time to functional recovery, poorer patient experience, and reduced hospital bed capacity and throughput (13, 14). The adjusted incremental prolongation of 4.20 days in LOS represents a large opportunity cost for healthcare systems with limited bed capacity.

Research addressing the economic and resource impact of PSIC in gynecologic surgery is exceptionally limited, underscoring the novelty and importance of our findings. Nonetheless, studies of the burden of disease due to healthcare associated infections (HAIs) and other postoperative infections report similar patterns.

A systematic review assessing the economic cost associated with SSI in low and middle income countries (LMICs) and high income European countries found that the extra cost related to SSIs was similar across studies, with additional costs in LMICs ranging from US$174 to US$29610 and in European countries from US$21 to US$34000. SSIs also significantly extended LOS by 3–35 days in LMICs. The substantial economic and resource burden of SSIs was consistent across settings, despite differences in healthcare system design and cost accounting methods (15). Our observed adjusted incremental cost increase of ¥8,960.74 and extended LOS of 4.20 days fall within this range, suggesting that the burden of PSIC in gynecologic surgery is comparable to infection burdens in other surgical areas.

Greco et al. demonstrated in a prospective multicenter cohort of adult cardiac operations that developing a postoperative bloodstream infection nearly doubled total hospital cost (86% increase), with costs rising from US$38200 before bloodstream infection to US$70900 after bloodstream infection (16). This figure aligns closely with our observation of increased cost following PSIC, of which bloodstream infection is a severe subset. Digiovine et al. (17) found that for primary nosocomial bloodstream infections in the intensive care unit, costs were 65% greater and LOS was more than 7.5 days longer for patients with bloodstream infection. These observations support the robustness of our findings and highlight the large impact of bloodstream infection.

Steiner et al. estimated that the occurrence of SSI after hysterectomy incurs an additional direct medical cost of approximately US$5000 (18). Although there is no direct research quantifying the additional direct medical cost of PSIC or bloodstream infection after hysterectomy, it is expected that, due to the systemic nature of bloodstream infection and other systemic infectious complications—which often require more intensive antimicrobial therapy, closer monitoring, and management of potential sepsis related complications—PSIC may represent an even greater economic burden than localized infections in gynecologic surgery patients.

The substantial economic burden identified in this study, an adjusted ¥8,960.74 in additional direct medical costs per PSIC case, highlights a strong potential for investment in prevention strategies. This amount, when multiplied across the thousands of gynecologic surgeries performed annually, represents significant potential savings for healthcare systems and payers. Furthermore, the additional 4.20 days of LOS associated with each PSIC case also represents considerable opportunity costs in bed availability and patient throughput, particularly in resource limited settings or during periods of high hospital occupancy (19).

From a cost effectiveness perspective, our findings suggest that even relatively expensive PSIC prevention bundles are likely to be justified. Evidence based interventions such as appropriate antimicrobial prophylaxis, chlorhexidine bathing, meticulous aseptic technique, and prompt removal of unnecessary invasive devices could yield substantial returns on investment (20, 21). Our previous studies, along with others, have shown that for gynecologic surgeries, particularly those involving the vaginal area, adequate preoperative vaginal preparation (such as vaginal scrubbing or douching with antiseptic solutions) can effectively reduce the risk of PSIC, including bloodstream infections (22, 23). This intervention may offer favorable economic viability when compared with the increased costs associated with treating PSIC.

Our results provide a foundation for larger evaluations of the economic burden of PSIC in gynecologic surgery. However, this study has limitations. First, as a retrospective analysis, we cannot exclude the possibility of unmeasured confounding factors affecting our findings, despite the use of PSM and doubly robust estimation. Second, our single center design may limit generalizability to other healthcare systems with different patient populations, clinical practices, and financial arrangements. Specifically, while these findings contextualize the burden under China’s emerging DRG/DIP payment models, the transferability to disparate global financing systems remains to be empirically tested. Third, we considered only direct medical costs related to the index hospitalization; we did not include indirect costs such as loss of productivity and caregiver burden, or post discharge costs such as readmissions and outpatient care, which may lead to underestimation of the total economic burden. Fourth, our syndromic definition of PSIC, wherein only 12.3% were microbiologically confirmed, likely encompasses severe non-infectious SIRS. While this captures the true economic reality of required interventions, it introduces clinical heterogeneity. Furthermore, the limited number of culture-positive cases precluded a formal sensitivity analysis restricted solely to microbiologically confirmed infections. Finally, it must be acknowledged that clinical decisions—such as ordering blood cultures or escalating antibiotics—are inherently influenced by provider behavior. Physicians may exhibit a lower threshold for initiating these interventions in sicker, highly complex, or oncologic patients, potentially introducing an ascertainment bias.

Future multicenter, prospective studies are needed to confirm external validity, provide tighter control for confounding factors, and examine long term and post discharge costs, including quality of life decrements. Cost effectiveness analyses of targeted prevention approaches, stratified by procedure specific and pathogen specific risk factors (for example, species and resistance profiles), will better inform resource allocation decisions. Including indirect costs such as loss of productivity, caregiver burden, and disability in societal perspective analyses would provide policymakers with a more comprehensive foundation for financing and quality improvement decisions.

## 5 Conclusion

This study, utilizing a doubly robust estimation framework following propensity score matching, provides valuable evidence that PSIC after gynecologic surgery independently and substantially increases direct medical costs and prolongs LOS. These findings underscore the critical importance of infection prevention and control measures in gynecologic surgical practice. Beyond improving patient outcomes, effective PSIC prevention strategies offer significant potential for cost savings and optimization of healthcare resource utilization. As healthcare systems increasingly focus on value-based care, targeting preventable complications with substantial economic impact, such as PSIC (including bloodstream infections), represents a high yield approach to simultaneously enhancing quality and controlling costs.

## Data Availability

The original contributions presented in the study are included in the article/supplementary material, further inquiries can be directed to the corresponding author.

## Glossary

ALB: Albumin
ALT: Alanine aminotransferase
AME: Average marginal effect
ASA: American Society of Anesthesiologists
AST: Aspartate aminotransferase
BMI: Body mass index
BSI: Bloodstream infection
CI: Confidence interval
CNY: Chinese Yuan
CREA: Creatinine
DRG/DIP: Diagnosis Related Groups/Diagnosis Intervention Packet
EMR: Electronic medical record
GFR: Glomerular filtration rate
GLM: Generalized linear model
HAIs: Healthcare-associated infections
HGB: Hemoglobin
ICU: Intensive care unit
IQR: Interquartile range
LMICs: Low-and middle-income countries
LOS: Length of stay
LPOS: Length of postoperative stay
LPPS: Length of preoperative preparation stay
PLT: Platelet
PSIC: Postoperative systemic infectious complications
PSM: Propensity score matching
SD: Standard deviation
SIRS: Systemic inflammatory response syndrome
SMD: Standardized mean difference
SSI: Surgical site infection
TBIL: Total bilirubin
WBC: White blood cell

## References

[1] Zimmermann JSM Sima RM Radosa MP Sima R‐M Radosa CG Ples L . Quality of life and sexual function in patients aged 35 years or younger undergoing hysterectomy for benign gynecologic conditions: a prospective cohort study. Int J Gynaecol Obstet. (2023) 160:548–53. doi: 10.1002/ijgo.14400, 35965372

[2] Han L. Current status and prospects of transvaginal natural orifice transluminal endoscopic surgery in gynecology. Chin J Pract Gynecol Obstet. (2019) 35:1300–4. doi: 10.19538/j.fk2019120102

[3] Peng C Zhou Y. Experience and reflections on laparoscopic hysterectomy for deep infiltrating endometriosis. Chin J Endosc Surg (Electron Ed). (2020) 13:77–80.

[4] Alkatout I O'Sullivan O Peters G Maass N. Expanding robotic-assisted surgery in gynecology using the potential of an advanced robotic system. Medicina (Kaunas). (2023) 60:53. doi: 10.3390/medicina60010053, 38256313 PMC10818539

[5] Allegranzi B Bagheri Nejad S Combescure C Nejad SB Graafmans W Attar H . Burden of endemic health-care-associated infection in developing countries: systematic review and meta-analysis. Lancet. (2011) 377:228–41. doi: 10.1016/S0140-6736(10)61458-4, 21146207

[6] Rashid N Begier E Lin KJ Yu H. Culture-confirmed *Staphylococcus aureus* infection after elective hysterectomy: burden of disease and risk factors. Surg Infect. (2020) 21:169–78. doi: 10.1089/sur.2019.043, 31580776

[7] Coello R Charlett A Wilson J Ward V Pearson A Borriello P. Adverse impact of surgical site infections in English hospitals. J Hosp Infect. (2005) 60:93–103. doi: 10.1016/j.jhin.2004.10.019, 15866006

[8] Wang YC Wu HY Luo CY Lin TW. Cardiopulmonary bypass time predicts early postoperative Enterobacteriaceae bloodstream infection. Ann Thorac Surg. (2019) 107:1333–41. doi: 10.1016/j.athoracsur.2018.11.020, 30552885

[9] Copeland-Halperin LR Emery E Collins D Liu C Dort J. Dogma without data: a clinical decision-making tool for postoperative blood cultures. Am Surg. (2018) 84:1339–44. doi: 10.1177/000313481808400849, 30185313

[10] Zimlichman E Henderson D Tamir O Franz C Song P Yamin CK . Health care-associated infections: a meta-analysis of costs and financial impact on the US health care system. JAMA Intern Med. (2013) 173:2039–46. doi: 10.1001/jamainternmed.2013.9763, 23999949

[11] Diekema DJ Beekmann SE Chapin KC Morel KA Munson E Doern GV. Epidemiology and outcome of nosocomial and community-onset bloodstream infection. J Clin Microbiol. (2003) 41:3655–60. doi: 10.1128/JCM.41.8.3655-3660.2003, 12904371 PMC179863

[12] Kaye KS Marchaim D Chen TY Baures T Anderson DJ Choi Y . Effect of nosocomial bloodstream infections on mortality, length of stay, and hospital costs in older adults. J Am Geriatr Soc. (2014) 62:306–11. doi: 10.1111/jgs.12634, 24438554 PMC4037885

[13] Perencevich EN Sands KE Cosgrove SE Guadagnoli E Meara E Platt R. Health and economic impact of surgical site infections diagnosed after hospital discharge. Emerg Infect Dis. (2003) 9:196–203. doi: 10.3201/eid0902.020232, 12603990 PMC2901944

[14] de Lissovoy G Fraeman K Hutchins V Murphy D Song D Vaughn BB. Surgical site infection: incidence and impact on hospital utilization and treatment costs. Am J Infect Control. (2009) 37:387–97. doi: 10.1016/j.ajic.2008.12.010, 19398246

[15] Monahan M Jowett S Pinkney T Brocklehurst P Morton DG Abdali Z . Surgical site infection and costs in low-and middle-income countries: a systematic review of the economic burden. PLoS One. (2020) 15:e0232960. doi: 10.1371/journal.pone.0232960, 32497086 PMC7272045

[16] Greco G Shi W Michler RE Meltzer DO Ailawadi G Hohmann SF . Costs associated with health care-associated infections in cardiac surgery. J Am Coll Cardiol. (2015) 65:15–23. doi: 10.1016/j.jacc.2014.09.079, 25572505 PMC4293042

[17] Digiovine B Chenoweth C Watts C Higgins M. The attributable mortality and costs of primary nosocomial bloodstream infections in the intensive care unit. Am J Respir Crit Care Med. (1999) 160:976–81. doi: 10.1164/ajrccm.160.3.9808145, 10471627

[18] Steiner HL Strand EA. Surgical-site infection in gynecologic surgery: pathophysiology and prevention. Am J Obstet Gynecol. (2017) 217:121–8. doi: 10.1016/j.ajog.2017.02.014, 28209490

[19] Graves N Halton K Lairson D. Economics and preventing hospital-acquired infection: broadening the perspective. Infect Control Hosp Epidemiol. (2007) 28:178–84. doi: 10.1086/510787, 17265399

[20] Anderson DJ Podgorny K Berríos-Torres SI Bratzler DW Dellinger EP Greene L . Strategies to prevent surgical site infections in acute care hospitals: 2014 update. Infect Control Hosp Epidemiol. (2014) 35:605–27. doi: 10.1086/676022, 24799638 PMC4267723

[21] Yokoe DS Anderson DJ Berenholtz SM Calfee DP Dubberke ER Ellingson KD . A compendium of strategies to prevent healthcare-associated infections in acute care hospitals: 2014 updates. Infect Control Hosp Epidemiol. (2014) 35:967–77. doi: 10.1086/677216, 25026611 PMC4223864

[22] Buppasiri P Chongsomchai C Wongproamas N Ounchai J Suwannachat B Lumbiganon P. Effectiveness of vaginal douching on febrile and infectious morbidities after total abdominal hysterectomy: a multicenter randomized controlled trial. J Med Assoc Thail. (2004) 87:16–23. 14971530

[23] Skeith AE Morgan DM Schmidt PC. Vaginal preparation with povidone-iodine or chlorhexidine before hysterectomy: a propensity score matched analysis. Am J Obstet Gynecol. (2021) 225:560.e1–9. doi: 10.1016/j.ajog.2021.08.035, 34473965

